# A Case of Convulsions Terminating Fatally, Caused by Ascaris Lumbricoides; Appearances Post Mortem

**Published:** 1848-04

**Authors:** William T. Winsor

**Affiliations:** Lexington, Missouri


Art. V.—*A Case of Convulsions terminating fatally, caused by Ascaris Lumbricoides; Appearances Post Mortem.* By William T. Winsor, M.D., of Lexington, Missouri.

A negro girl, aged four years, was taken with convulsions, which lasted some minutes, and were followed by stupor and insensibility. The symptoms on visiting the patient were as follows: Pulse extremely vacillating, at times small, frequent and feeble, and again full and slow, but always compressible; skin a little above the natural temperature and dry; breathing rapid and laborious; eyes tightly closed, and condition of the pupils not determined. The child had been previously healthy, and immediately before the attack manifested no signs of indisposition except complaining of pain in the head; but for a month previously she had manifested a propensity to eat dirt, which nothing had been found to correct.

Conceiving the nervous or cerebral irritation to be purely sympathetic, and that the primary local irritation was in the alimentary canal, and probably caused by the presence of lumbrici, it was thought proper to administer castor oil and spirits of turpentine freely, and to use frictions with the latter over the epigastric and umbilical regions; a blister plaster was also applied to the back of the neck, and laxative enemata employed. This practice was continued for several hours without any effect upon the bowels or amendment in the symptoms, when venesection, calomel and some of the more drastic purgatives were resorted to; but all proved ineffectual. The convulsions returned at intervals, alternating with a lethargic condition from which she could not easily be roused. She died in thirty hours from the commencement of the convulsions. Permission being given to make a *post mortem* examination, the following apperances were presented by the *sectio cadaveris*, twenty-four hours after death:

*Brain.*—No evidence of inflammation in the meninges. Serous effusion on the arachnoid; great vascular turgescence of the entire cerebral mass; sinuses full—the great longitudinal and lateral ones distended as if by injection; traces of pus between the convolutions and accompanying the ramifications of the vessels on the surface of the brain and between the arachnoid and pia mater. Consistence of the nervous mass less than natural, amounting to incipient softening.

*Abdomen.*—The intestinal tube contained little else than lumbrici throughout its entire extent, except the colon. *Seven distinct invaginations* were discovered in the small intestines. The first was twelve inches below the duodenum, the last was within six inches of the cœcum. The intussusceptions were all *progressive*, and the invaginated portion of some exceeded two inches in length. No symptoms of inflammation were present in the bowel or its mucous lining, nor even hyperæmia. In fact, it was exsanguine, and distended with flatus; and between each invagination, knots of worms could be felt, and even seen through the expanded and translucent portions when held up to the light. A large worm had found its way into the appendix vermiformis, but beyond this none were found. Mesenteric glands enlarged; gall-bladder full of vitiated bile; no obstruction in the gall-duct; liver of a healthy color, but extremely tough, and could not be broken down by the thumb and finger.—No examination of the thorax.

January, 1848.

